# [Retracted] lncRNA‑MIAT regulates cell biological behaviors in gastric cancer through a mechanism involving the miR‑29a‑3p/HDAC4 axis

**DOI:** 10.3892/or.2025.8902

**Published:** 2025-04-22

**Authors:** Yanfeng Li, Kuan Wang, Yuzhe Wei, Qiang Yao, Qifan Zhang, Hongyan Qu, Guanyu Zhu

Oncol Rep 38: 3465–3472, 2017; DOI: 10.3892/or.2017.6020

Following the publication of the above article, a concerned reader drew to the Editor's attention that, regarding the cell invasion assay data shown in Fig. 2B on p. 3469, the ‘si-NC (SGC7901)’ and ‘si-NC (MGC803)’ data panels appeared to show the same data, albeit the panels were presented in a different orientation with respect to each other (rotated through 180°). Moreover, the data in question had apparently already been submitted and published in an article in the journal *Oncology Letters* written by different authors, although from the same hospital.

After having performed an internal investigation of these data in the Editorial Office, the Editor of *Oncology Reports* has confirmed the legitimacy of the reader's concerns. Therefore, the Editor has decided that this article should be retracted from the publication on the grounds of an overall lack of confidence in the data, and given that the same data appeared in an unrelated article in a different journal. The authors were asked for an explanation to account for these concerns, but the Editorial Office did not receive a reply. The Editor sincerely apologizes to the readership for any incovenience caused, and we thank the reader for bringing this matter to our attention.

